# Mathematical models of SIR disease spread with combined non-sexual and sexual transmission routes

**DOI:** 10.1016/j.idm.2016.12.003

**Published:** 2017-01-11

**Authors:** Joel C. Miller

**Affiliations:** a Monash University, School of Mathematical Sciences, Melbourne 3800, Australia; b Institute for Disease Modeling, Bellevue, WA, USA

## Abstract

The emergence of Zika and Ebola demonstrates the importance of understanding the role of sexual transmission in the spread of diseases with a primarily non-sexual transmission route. In this paper, we develop low-dimensional models for how an SIR disease will spread if it transmits through a sexual contact network and some other transmission mechanism, such as direct contact or vectors. We show that the models derived accurately predict the dynamics of simulations in the large population limit, and investigate ${ℛ}_{0}$ and final size relations.

## 1 Introduction

The recent emergence of Ebola (Christie et al., 2015, Mate et al., 2015) and Zika (Foy et al., 2011, Musso et al., 2015) demonstrates that diseases which spread primarily through other means can also have a sexual component to their spread.

Zika is a mosquito-borne virus which can cause birth defects if a pregnant woman is infected (Oliveira Melo et al., 2016, Ventura et al., 2016). Although more is being learned, it appears that Zika causes self-limiting infections (Duffy et al., 2009) and it is likely that infected individuals recover with immunity (Lessler et al., 2016).

Ebola is a directly transmitted disease which causes extreme morbidity and mortality (Baron et al., 1983, Emond et al., 1977, Heymann et al., 1980). It is spread through direct contact with bodily fluids from an infected individual. Individuals who survive appear to gain immunity (McElroy et al., 2015).

For both Zika and Ebola, evidence suggests that sexual transmission is possible (Christie et al., 2015, Foy et al., 2011, Mate et al., 2015, Musso et al., 2015). Further, it appears that individuals may be able to transmit the viruses sexually even after they appear to no longer be infectious through the usual mechanisms (Mackay and Arden, 2015, Deen et al., 2015, Nicastri et al., 2016).

To better predict the spread of such diseases, we need to capture these different transmission modes into a model. Unfortunately, many existing mathematical models of disease spread through networks require a large number of equations (Kiss et al., 2017, Miller and Kiss, 2014). However, in the specific case of Susceptible–Infected–Recovered (SIR) diseases such as Ebola and Zika, a low dimensional model, the Edge-based compartmental model, exists which can capture a diverse set of assumptions about the network structure (Miller et al., 2012, Miller and Volz, 2013). However, it is structurally very different from the usual models used for other transmission mechanisms. Consequently it is not immediately obvious that we can combine the different transmission models into a single low-dimensional mathematical model.

Our goal in this paper is to develop simple mathematical models which capture a range of different transmission mechanisms combined with a sexual mode of transmission, and to provide enough examples to show how various assumptions can be combined into simple, low-dimensional models.

We begin by revisiting existing models for an SIR disease spreading through mass-action mixing and for an SIR disease spreading through a sexual contact network. We explore a number of models of the spread of an SIR disease through a sexual contact network combined with another transmission mechanism. In all of the models we build, we assume that the sexually infectious period lasts longer than the period of infectiousness through the other mechanism. It is straightforward to modify this assumption. We divide the models presented into two broad classes:

• We consider a random static sexual network (a “configuration model” network (Newman, 2003)) combined with some other transmission model, in particular
  – mass action mixing,
  – vector-borne transmission,
  – or social contact network.
• We then consider a simple mass-action transmission model combined with a more complex sexual transmission network including:
  – dynamic partnership changes
  – preferential mixing.

## 2 Models with configuration model networks

Throughout we assume that *S*, *I*, and *R* (and any subdivisions of these classes) represent the proportion of the population in the susceptible, infected, or recovered state. We assume the outbreak is initialized with a fraction *ρ* of the population chosen uniformly at random and infected at time t=0.

### 2.1 The standard models

We briefly review the mass-action SIR model (Kermack & McKendrick, 1927) and the Edge-based compartmental model (Miller et al., 2012, Miller, 2011). Although these models appear structurally different, a simple change of variables shows that they are closely related. This close relation will allow us to combine mixtures of the models.

#### 2.1.1 The mass-action model

**Fig. 1:**
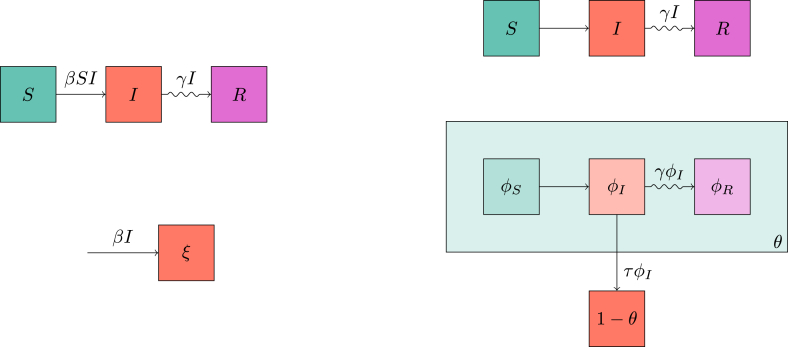
Flow diagrams leading to systems (1), (2), and (3). (top left) A flow diagram demonstrating the standard model of mass action SIR dynamics. (right) Two flow diagrams showing the EBCM model of network SIR dynamics. (bottom left) A flow diagram, that when combined with the diagram above it allows for a system of equations similar to the EBCM model.

We begin with the mass-action SIR model. An infectious individual transmits infectious doses as a Poisson process with rate *β*. Each infectious dose is received by an individual randomly chosen from the population. If the recipient is susceptible, then she becomes infectious. Infected individuals recover as a one-step Poisson process with rate *γ*. Once recovered they are immune to future infection. The diagram in the top-left of Fig. 1 leads us to the equations

(1a) $$\dot{S}=-\beta IS$$

(1b) $$\dot{I}=\beta IS-\gamma I$$

(1c) $$\dot{R}=\gamma I$$

with initial conditions

$$\begin{array}{l} S\left(0\right)=1-\rho \\ I\left(0\right)=\rho \\ R\left(0\right)=0. \end{array}$$

**Fig. 2:**
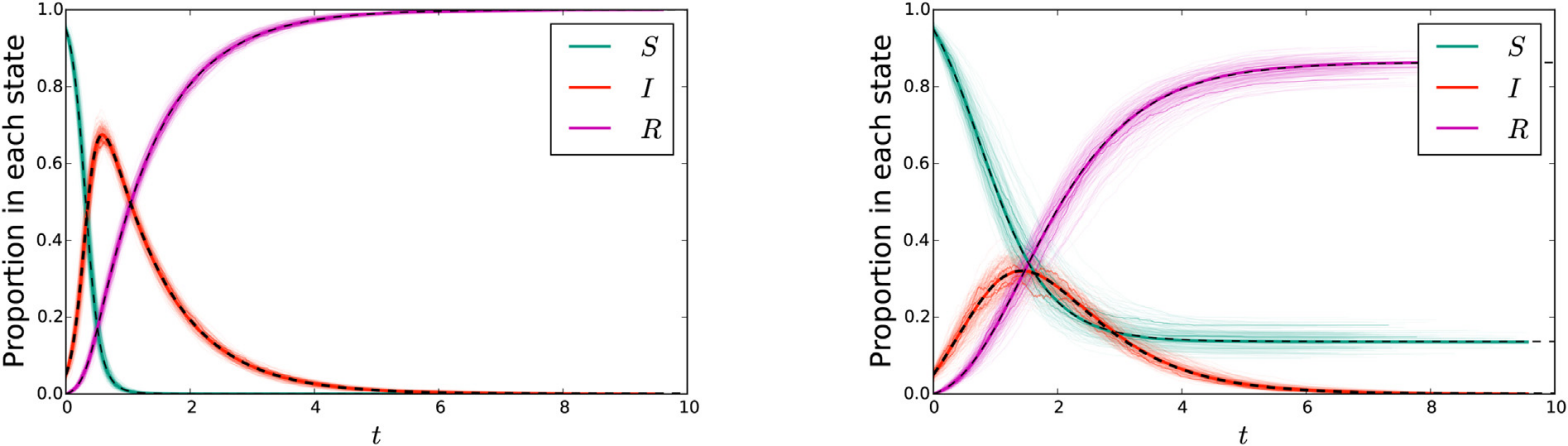
(left) A comparison of stochastic agent-based simulation with ODE solution for the mass action model (1) or (3). (right) A comparison of stochastic agent-based simulation with ODE solution for the network-based model (2). In both cases, dashed curve denotes the solution to the ODE. A cloud made up of 200 simulations in populations of $10^{3}$ individuals is shown lightly in color, with 3 of these simulations highlighted. An additional simulation in a population of $10^{5}$ individuals is shown in darker color, almost exactly matching the ODE solution. In the mass action model, the parameters are β=10 and γ=1. The network has P(2)=P(4)=0.5, so $\psi \left(x\right)=\left(x^{2}+x^{4}\right)/2$. The parameters are τ=2 and γ=1.

Fig. 2 shows that the solutions of system (1) accurately predicts the outcome of stochastic simulation in the large-population limit. The fit is excellent. Indeed, the equations correctly predict the large-population limit of the simulations. The convergence can be understood through the results of (Kurtz, 1971).

#### 2.1.2 Edge-based compartmental model

For the simplest network-based model, we assume that a function P(k) is known which gives the probability a randomly chosen individual has *k* partners (its *degree*). If we assume that partners are randomly chosen, then the probability a random partner of a random individual has degree *k* is $P_{n}\left(k\right)=kP\left(k\right)/\langle K\rangle$ where 〈K〉=∑kP(k) is the average degree.

This model differs from the mass-action model by assuming individuals transmit only through partnerships which do not change. Thus as an individual transmits to its partners, the number of available susceptible partners is reduced. The transmissions occur as Poisson processes with rate *τ*. As before, if the recipient is susceptible he becomes infected, and eventually recovers to an immune state with rate *γ*. The two diagrams on the right of Fig. 1 leads us to the Edge-based compartmental model (EBCM) (Miller et al., 2012) for a population with arbitrary initial fraction *ρ* infected (Miller et al., 2012, Miller, 2011, Miller, 2014, Kiss et al., 2017, chapter 6)

(2a) $$\dot{\theta }=-\tau \phi_{I}$$

(2b) $$\phi_{I}=\theta -\left(1-\rho \right)\frac{\psi \text{'}\left(\theta \right)}{\langle K\rangle }-\frac{\gamma }{\tau }\left(1-\theta \right)$$

(2c) S=(1−ρ)ψ(θ)

(2d) I=1−S−R

(2e) $$\dot{R}=\gamma I$$

with initial conditions

$$\begin{array}{l} \theta \left(0\right)=1\\ R\left(0\right)=0 \end{array}$$

where $\psi \left(x\right)=\sum_{k}P\left(k\right)x^{k}$ is the probability generating function of the degree distribution. Here θ(t) represents the probability that at time *t* a randomly chosen partner *v* of a randomly chosen individual *u* has not transmitted infection to *u* and $\phi_{I}$ represents the probability that *v* is infected and has not transmitted infection to *u*. These equations have been proven correct for random networks of given degree distribution in the infinite population limit (Janson, Luczak, & Windridge, 2014), and under stronger assumptions by (Decreusefond, Dhersin, Moyal, & Tran, 2012). See also (Ball & Neal, 2008) which derived a large system of equations that can be shown to be equivalent to system (2) (Miller & Kiss, 2014).

We briefly outline a derivation of these equations, glossing over some more subtle details. We first note that the probability a degree *k* individual is susceptible is $\theta^{k}$. Thus taking a weighted average of $\theta^{k}$, we conclude that $S\left(t\right)=\sum_{k}P\left(k\right)\theta {\left(t\right)}^{k}=\psi \left(\theta \right)$. That I=1−S−R and $\dot{R}=\gamma I$ is straightforward. So it remains to find an equation for *θ*. We divide *θ* into three parts, $\theta =\phi_{S}+\phi_{I}+\phi_{R}$ based on the status of *v*.

• $\phi_{S}$: the probability that the partner *v* of *u* is susceptible (and has not transmitted to *u*).
• $\phi_{I}$: the probability that *v* is infected and has not transmitted to *u.*
• $\phi_{R}$: the probability that *v* is recovered and did not transmit to *u.*

We can find $\phi_{S}$ in terms of *θ* starting with the observation *v* has degree *k* with probability $P_{n}\left(k\right)=kP\left(k\right)/\langle K\rangle$ which accounts for the fact that a random partner can be expected to have higher degree than a random individual (Feld, 1991). Following the derivation of *S*, we get $\phi_{S}=\left(1-\rho \right)\sum kP\left(k\right)\theta^{k-1}/\langle K\rangle$ where $\theta^{k-1}$ is the probability that none of *v*'s other partners have transmitted to it (we can assume *u* has not). Thus

$$\phi_{S}=\left(1-\rho \right)\psi^{\prime }\left(\theta \right)/\langle K\rangle .$$

We can find $\phi_{R}$ in terms of *θ* starting with the observation that ${\dot{\phi }}_{R}=\gamma \phi_{I}$. Then ${\dot{\phi }}_{R}$ and $\dot{\theta }$ are proportional. Using θ(0)=1 and $\phi_{R}\left(0\right)=0$, we can show that

$$\phi_{R}=\gamma \left(1-\theta \right)/\tau .$$

Thus

$$\begin{array}{c} \phi_{I}=\theta -\phi_{S}-\phi_{R}\\ =\theta -\left(1-\rho \right)\frac{\psi^{\prime }\left(\theta \right)}{\langle K\rangle }-\frac{\gamma }{\tau }\left(1-\theta \right). \end{array}$$

More details (including a subtle argument explaining that we can ignore transmissions from *u* to *v*) are found in (Miller et al., 2012) and (Kiss et al., 2017, chapter 6).

The right hand side of Fig. 2 compares stochastic simulation with solutions of system (2). The fit is excellent.

#### 2.1.3 Model similarity

The two models (1) and (2) appear quit dissimilar structurally, which would make it difficult to derive a hybrid model. However, we can modify the mass action system (1) making it easier to combined the models.

We define $\xi \left(t\right)=\beta \int_{0}^{t}I\left(\hat{t}\right)\;\text{d}\hat{t}$ to be the expected number of transmissions a random individual has received by time *t*. Using an integrating factor we see

$$\dot{S}+\beta IS=0$$

becomes

$$\frac{\text{d}}{\text{d}t}\left(Se^{\beta \int_{0}^{t}I\;\text{d}\hat{t}}\right)=0\;.$$

From this it follows that

$$S\left(t\right)=S\left(0\right)e^{-\xi \left(t\right)}=\left(1-\rho \right)e^{-\xi \left(t\right)}\;.$$

From the definition of *ξ* we conclude $\dot{\xi }=\beta I$. Because $\dot{R}=\gamma I$, and R(0)=ξ(0)=0, we can show that R=γξ/β. This leads us to

(3a) $$S=\left(1-\rho \right)e^{-\xi }$$

(3b) $$I=1-S-R=1-\left(1-\rho \right)e^{-\xi }-\frac{\gamma \xi }{\beta }$$

(3c) $$R=\frac{\gamma \xi }{\beta }$$

(3d) $$\dot{\xi }=\beta I=\beta \left[1-\left(1-\rho \right)e^{-\xi }-\frac{\gamma \xi }{\beta }\right]$$

with the initial condition

$$\xi \left(0\right)=0\;.$$

The variable *ξ* plays a similar role to *θ* [more precisely, $e^{-\xi }$ plays the same role as ψ(θ)].

We provide a direct derivation of system (3), for which we consider both diagrams on the left of Fig. 1. We interpret *ξ* as the expected number of transmissions an individual has received. An individual is susceptible if she has received no transmissions and was not initially infected. If the expected number of transmissions is *ξ*, and each is randomly assigned, the probability of escaping transmission is $e^{-\xi }$. Thus $S=\left(1-\rho \right)e^{-\xi }$. To find *ξ*, we see that the rate of receiving transmissions is βI, so $\dot{\xi }=\beta I$. Then *I* and *R* follow as before, and we have a direct derivation of system (3) without going through system (1) first.

#### 2.1.4 ${ℛ}_{0}$

An important quantity of mathematical epidemiology is the basic reproductive number ${ℛ}_{0}$. This is often defined to be the expected number of infections caused when a single infected individual is introduced into a fully susceptible population. When the population exhibits heterogeneity, or partnerships have non-negligible duration, this definition breaks down. If we want ${ℛ}_{0}$ to accurately capture the transmission in the early stages of an epidemic, then its definition must account for the fact that the typical infected individual early in an epidemic may not be a typical individual in the population, and we must account for the fact that an individual cannot reinfect his infector. In defining ${ℛ}_{0}$ we must account for what the typical infected individuals looks like once the system settles down rather than what the typical introduced infected individual looks like (Diekmann et al., 1990, Diekmann et al., 2009, Diekmann et al., 2012).

##### 2.1.4.1 Mass-action model

In the mass-action model, we assume infected individuals recover with rate *γ*, thus having an average infection duration of 1/γ. All infected individuals transmit at rate *β* and the recipient is chosen uniformly at random from the population. Early in the epidemic, all transmissions reach a susceptible individual, thus causing infection. The expected number of infections caused by an individual infected early in the epidemic is thus

$${ℛ}_{0}=\beta /\gamma .$$

##### 2.1.4.2 Network-based model

In the network model, transmissions go only to direct partners of the infected individuals, and a non-negligible proportion of these transmissions may go to an individual’s infector, thus having no effect. Early in the epidemic, an individual is chosen by the disease with probability proportional to their degree. So the early infections have degree *k* with probability $P_{n}\left(k\right)=kP\left(k\right)/\langle K\rangle$. The probability of transmitting along a partnership prior to recovery is τ/(τ+γ). Thus the expected number of transmissions is

$$\begin{array}{l} {ℛ}_{0}=\sum_{k}\frac{kP\left(k\right)}{\langle K\rangle }\left(k-1\right)\frac{\tau }{\tau +\gamma }\\ =\frac{\tau \langle K^{2}-K\rangle }{\left(\tau +\gamma \right)\langle K\rangle }\;. \end{array}$$

#### 2.1.5 Final size relation

There is a well-known final size relation for the mass-action model (1) linking the number of infections to ${ℛ}_{0}$. The usual derivation involves dividing $\dot{I}$ by $\dot{S}$, finding *I* as a function of *S* and then finding the values of *S* for which I=0. We find that system (3) makes this more direct. We simply note that at equilibrium $\dot{\xi }=0$, and solve to get $\xi \left(\infty \right)/{ℛ}_{0}=1-\left(1-\rho \right)e^{-\xi \left(\infty \right)}$ Substituting $R=\xi /{ℛ}_{0}$, we conclude

$$R\left(\infty \right)=1-\left(1-\rho \right)e^{-{ℛ}_{0}R\left(\infty \right)}$$

which is the final size relation. The easiest way to find a solution is to iterate, starting with a guess R(∞)=0.

There is a similar final size relation (Newman, 2002). The usual derivation is based on directly deriving a consistency relation without having a dynamic system of equations. We derive it from the EBCM equation (2a), (2b), (2c), (2d), (2e). Taking $\phi_{I}=0$, we find

(4) $$\theta \left(\infty \right)=\left(1-\rho \right)\frac{\psi^{’}\left(\theta \left(\infty \right)\right)}{\langle K\rangle }=\frac{\gamma \left(1-\theta \left(\infty \right)\right)}{\tau }\;.$$

We also solve this through iteration, starting with θ(0)=1.

Both the network and mass action final size relations can be intepreted as calculating the number of transmissions occurring given the number of infected individuals, and then in turn calculating the number of infected individuals assuming that those transmissions are randomly distributed. In general, this method can be used to calculate all final size relations of which we are aware.

It is instructional to recognize that with appropriate initial conditions the iteration corresponds directly to solving the dynamics of a particular discrete-time (Reed-Frost) disease model.^1^ Each iteration gives the successive generation's size. Thus iterating until convergence gives the result as the number of generations tends to ∞. This is discussed in more detail in (Pellis, Ferguson, & Fraser, 2008) (see also (Ludwig, 1975) and some cautionary discussion in (Fennell, Melnik, & Gleeson, 2016)).

If, however, the timing of *v*'s infection affects whether it would transmit to *u* (as may happen if there are seasonal effects or if the number of other infections in the same timestep somehow influences *v*'s likelihood of transmitting), then we cannot derive a final size relation in the implicit way we have done it here. Instead, we must solve the dynamical equations. This represents the fact that we cannot predict the number of transmissions *v* has caused until we know the time at which *v* becomes infected.

### 2.2 The basic combined model

Our first new model assumes two modes of transmission: one is mass-action, while the other is through a sexual contact network. We take *θ* to be the probability a random sexual partner of an individual *u* has not transmitted to *u*, while we define ξ(t) to be the expected number of transmissions *u* has received by time *t* through mass action transmission.

We assume there are four possible statuses. Individuals begin susceptible (*S*). Through either sexual or mass-action interactions, they become infectious. The infectious phase occurs in two stages. The initial stage ($I_{1}$) is infectious through both mass action and sexual contact. The second stage ($I_{2}$) is infectious only through sexual contact. They finally reach an immune recovered state (*R*). The symbols *S*, $I_{1}$, $I_{2}$, and *R* denote both the stage and the fraction of individuals in that stage, so $S+I_{1}+I_{2}+R=1$.

**Fig. 3:**
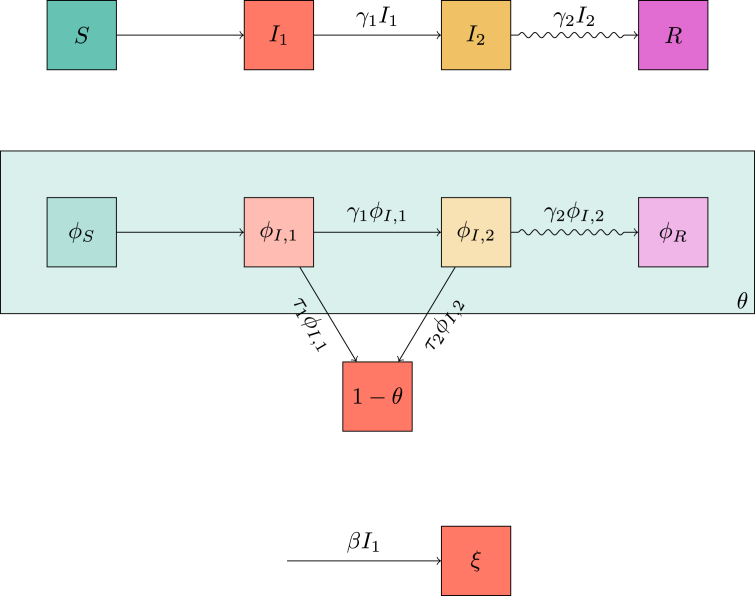
Flow diagrams leading to system (5). The top diagram shows transitions of individuals between the states. The middle diagram shows transitions of the status of a partner *v* of a randomly chosen individual *u* as well as whether *v* has transmitted to *u* (all ignoring transmissions to *u* to *v*). The bottom diagram shows the change in *ξ* and how it relates to $I_{1}$. These diagrams are related together by the dependence of the flux into *ξ* on $I_{1}$ and on the fact that we can express *S* and $\phi_{S}$ in terms of *ξ* and *θ*.

The per-individual transition rate from $I_{1}$ to $I_{2}$ is $\gamma_{1}$, and the per-individual transition rate from $I_{2}$ to *R* is $\gamma_{2}$. The mass action transmission rate is *β*, while the per-partnership transmission rate during $I_{1}$ is $\tau_{1}$ and during $I_{2}$ is $\tau_{2}$. Fig. 3 shows the corresponding flow diagrams for the combined model.

We can find $S\left(t\right)=\left(1-\rho \right)e^{-\xi \left(t\right)}\psi \left(\theta \left(t\right)\right)$, representing the fact that *S* is the probability an individual was not initially infected, has not been infected through mass-action transmission, and has not been infected through sexual transmission. Using the top diagram in Fig. 3 then shows that ${\dot{I}}_{2}=\gamma_{1}I_{1}-\gamma_{2}I_{2}$ and $\dot{R}=\gamma_{2}I_{2}$. Conservation of probability gives $I_{1}=1-S-I_{2}-R$. We are only missing equations for *θ* and *ξ*.

As before the equation for *ξ* is simply $\dot{\xi }=\beta I_{1}$. The equation for *θ* is very similar to before. We have $\dot{\theta }=\tau_{1}\phi_{I,1}-\tau_{2}\phi_{I,2}$. We will use $\phi_{I,1}=\theta -\phi_{S}-\phi_{I,2}-\phi_{R}$. We can again find $\phi_{S}$ explicitly, the only change is that there is an additional factor $e^{-\xi }$ accounting for mass-action transmissions: $\phi_{S}=\left(1-\rho \right)e^{-\xi }\psi^{\prime }\left(\theta \right)/\langle K\rangle$. We cannot explicitly find $\phi_{I,2}$ and $\phi_{R}$, but we have differential equations for them: ${\dot{\phi }}_{I,2}=\gamma_{1}\phi_{I,1}-\left(\tau_{2}+\gamma_{2}\right)\phi_{I,2}$ and ${\dot{\phi }}_{R}=\gamma_{2}\phi_{I,2}$. The final system is

(5a) $$S=\left(1-\rho \right)e^{-\xi }\psi \left(\theta \right)$$

(5b) $$I_{1}=1-S-I_{2}-R$$

(5c) $${\dot{I}}_{2}=\gamma_{1}I_{1}-\gamma_{2}I_{2}$$

(5d) $$\dot{R}=\gamma_{2}I_{2}$$

(5e) $$\dot{\xi }=\beta I_{1}$$

(5f) $$\dot{\theta }=-\tau_{1}\phi_{I,1}-\tau_{2}\phi_{I,2}$$

(5g) $$\phi_{S}=\frac{\left(1-\rho \right)e^{-\xi }\psi^{\prime }\left(\theta \right)}{\langle K\rangle }$$

(5h) $$\phi_{I,1}=\theta -\phi_{S}-\phi_{I,2}-\phi_{R}$$

(5i) $${\dot{\phi }}_{I,2}=\gamma_{1}\phi_{I,1}-\left(\gamma_{2}+\tau_{2}\right)\phi_{I,2}$$

(5j) $${\dot{\phi }}_{R}=\gamma_{2}\phi_{I,2}\;.$$

If we take $\tau_{2}=0$ or $\gamma_{2}=\infty$, then assuming a randomly introduced infection, this model is equivalent to the model considered by (Ball & Neal, 2008), although it is much more compact. The equivalence follows from techniques of (Miller & Kiss, 2014).

**Fig. 4:**
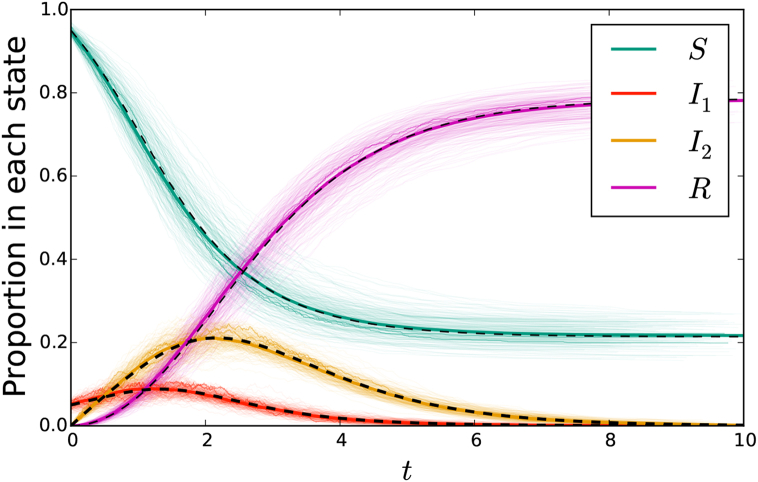
A comparison of simulation with solutions to system (5) for network-based transmission combined with mass-action transmission. The colored cloud consists of 200 simulations in a population of $10^{3}$ individuals, with three highlighted. A darker solid colored curve shows a simulation in a population of $10^{5}$. This is almost exactly overlain by the solution to the ODE system (5) shown in a black dashed curve. The parameters are β=1, $\tau_{1}=1$, $\gamma_{1}=3$, $\tau_{2}=1/2$, and $\gamma_{2}=1$. The function *ψ* takes the form $\left(x^{2}+x^{4}\right)/2$.

Fig. 4 compares simulated epidemics in large populations with predictions from system (5).

#### 2.2.1 ${ℛ}_{0}$

In deriving ${ℛ}_{0}$, we separate the individuals by their “generation”, that is, the length of the transmission chain from the initial case. The relation between one generation and the next is found through a matrix multiplication. The result quickly settles down to a multiple of the dominant eigenvector, with ${ℛ}_{0}$ being the dominant eigenvalue. We demonstrate this calculation using two different approaches, because each will be useful in the following models. The approaches track different variables, resulting in different matrices.

Before giving the derivations, we note that the probability a sexual partnership will transmit during the first infectious stage if one individual is infected is $\tau_{1}/\left(\tau_{1}+\gamma_{1}\right)$, and if transmission does not occur in the first stage the probability of transmission in the second stage is $\tau_{2}/\left(\tau_{2}+\gamma_{2}\right)$. Combining this, the full probability of sexual transmission within a partnership is $T_{\text{se}}=1-\left(\gamma_{1}\gamma_{2}\right)/\left(\tau_{1}+\gamma_{1}\right)\left(\tau_{2}+\gamma_{2}\right)$.

The expected number of mass-action transmissions from a single infected individual is $R_{\text{ma}}=\beta /\gamma_{1}$. The expected number of sexual transmissions from an individual infected through the mass-action route is $R_{\text{se}|\text{ma}}=T_{\text{se}}\langle K\rangle$ and the expected number of sexual transmissions from an individual infected through the sexual route is $R_{\text{se}|\text{se}}=T_{\text{se}}\langle K^{2}-K\rangle /\langle K\rangle$.

##### 2.2.1.1 First derivation

For our first derivation, we distinguish individuals based on whether they were infected through a mass-action transmission or through a sexual transmission. The distinction is needed because early in the epidemic individuals infected through a mass-action transmission have on average 〈K〉 susceptible sexual partners, while those infected through a sexual transmission have on average $\langle K^{2}-K\rangle /\langle K\rangle$ susceptible sexual partners. The number of mass-action transmissions caused by each class of individual is the same.

We define $N_{\text{ma}}\left(g\right)$ and $N_{\text{se}}\left(g\right)$ to be the number of individuals in generation *g* which were infected through mass-action and sexual transmissions respectively. Then

$$\left(\begin{array}{c} N_{\text{ma}}\left(g+1\right)\\ N_{\text{se}}\left(g+1\right) \end{array}\right)=\left(\begin{array}{cc} R_{\text{ma}} & R_{\text{ma}}\\ R_{\text{se}|\text{ma}} & R_{\text{se}|\text{se}} \end{array}\right)\left(\begin{array}{c} N_{\text{ma}}\left(g\right)\\ N_{\text{se}}\left(g\right) \end{array}\right)\;.$$

The dominant eigenvalue is

$$\begin{array}{c} R_{0}=\frac{R_{\text{ma}}+R_{\text{se}|\text{se}}+\sqrt{{\left(R_{\text{ma}}+R_{\text{se}|\text{se}}\right)}^{2}-4\left(R_{\text{ma}}R_{\text{se}|\text{se}}-R_{\text{se}|\text{ma}}R_{\text{ma}}\right)}}{2}\\ =\frac{R_{\text{ma}}+R_{\text{se}|\text{se}}+\sqrt{{\left(R_{\text{ma}}-R_{\text{se}|\text{se}}\right)}^{2}+4R_{\text{se}|\text{ma}}R_{\text{ma}}}}{2}. \end{array}$$

##### 2.2.1.2 Second derivation

An alternate pair of variables that could be considered is the number of infected individuals and the number of discordant (SI) partnerships. We use N(g) to denote the number infected in generation *g* and y(g) to be the number of discordant partnerships in generation *g*. Then N(g+1) is the sum of the number of infections caused by the mass action route, $R_{\text{ma}}N\left(g\right)$ and the number caused by sexual transmission $T_{\text{se}}y\left(g\right)=R_{\text{se}|\text{ma}}y\left(g\right)/\langle K\rangle$. Similarly the number of new discordant partnerships is the sum of the number created through the mass-action route $\langle K\rangle R_{\text{ma}}N\left(g\right)$ and the sexual route $\left[T_{\text{se}}\langle K^{2}-K\rangle /\langle K\rangle \right]y\left(g\right)=R_{\text{se}|\text{se}}y\left(g\right)$. Thus

$$\left(\begin{array}{c} N\left(g+1\right)\\ y\left(g+1\right) \end{array}\right)=\left(\begin{array}{cc} R_{\text{ma}} & R_{\text{se}|\text{ma}}/\langle K\rangle \\ R_{\text{ma}}\langle K\rangle & R_{\text{se}|\text{se}} \end{array}\right)\left(\begin{array}{c} N\left(g\right)\\ y\left(g\right) \end{array}\right)\;.$$

The eigenvalues of this matrix are the same as before.

##### 2.2.1.3 Approximations of ${ℛ}_{0}$

We highlight the fact that ${ℛ}_{0}$ is not simply a sum of $R_{\text{ma}}$ and $R_{\text{se}|\text{se}}$. We will discuss this more in the vector-borne disease model.

It is useful to recognize that by expanding the square root as a binomial series, our expression for ${ℛ}_{0}$ can be approximated as

$${ℛ}_{0}=\frac{R_{\text{ma}}+R_{\text{se}|\text{se}}+|R_{\text{ma}}-R_{\text{se}|\text{se}}|}{2}+\frac{R_{\text{se}|\text{ma}}R_{\text{ma}}}{\left|R_{\text{ma}}-R_{\text{se}|\text{se}}\right|}+O\left(\frac{{\left(R_{\text{se}|\text{ma}}R_{\text{ma}}\right)}^{2}}{|R_{\text{ma}}-R_{\text{se}|\text{se}}{|}^{3}}\right).$$

If either $R_{\text{ma}}$ or $R_{\text{se}|\text{se}}$ is very small, then this approximates as $R_{\text{se}|\text{se}}$ or $R_{\text{ma}}$ respectively If $R_{\text{ma}}R_{\text{se}|\text{ma}}\neq 0$, then ${ℛ}_{0}$ is larger than the maximum of $R_{\text{ma}}$ and $R_{\text{se}}$.

However, if $R_{\text{ma}}\approx R_{\text{se}|\text{se}}$, this approximation breaks down. Then writing $R_{\text{ma}}=R_{\text{se}|\text{se}}+\varepsilon$, we find

$$\begin{array}{c} R_{0}=\frac{2R_{\text{se}|\text{se}}+\varepsilon +\sqrt{{\left(2R_{\text{se}|\text{se}}+\varepsilon \right)}^{2}-4R_{\text{se}|\text{se}}^{2}-4\varepsilon R_{\text{se}|\text{se}}+4R_{\text{se}|\text{ma}}R_{\text{ma}}}}{2}\\ =R_{\text{se}|\text{se}}+\frac{\varepsilon +\sqrt{4R_{\text{se}|\text{ma}}R_{\text{ma}}+\varepsilon^{2}}}{2}\\ \approx R_{\text{se}|\text{se}}+\sqrt{R_{\text{se}|\text{ma}}R_{\text{ma}}}\;. \end{array}$$

#### 2.2.2 Final size relation

For this model, we can derive a consistency relation which leads to the final size. We can do this in two ways. We first show an indirect derivation by integrating the full dynamical equations. However, these equations can be derived directly through a mechanistic argument, and so we show this argument as well.

##### 2.2.2.1 Indirect derivation

We can derive a final size relation. We take t→∞ and note that all the variables corresponding to active infectious states must go to zero. Then

$$\begin{array}{l} S\left(\infty \right)=\left(1-\rho \right)e^{-\xi \left(\infty \right)}\psi \left(\theta \left(\infty \right)\right)\\ \phi_{S}\left(\infty \right)=\left(1-\rho \right)e^{-\xi \left(\infty \right)}\frac{\psi^{’}\left(\theta \left(\infty \right)\right)}{\langle K\rangle }\;. \end{array}$$

We use this to express ξ(∞) and θ(∞) in terms of ξ(∞) and θ(∞). This yields an implicit relation to solve.

We first find ξ(∞). We have $R\left(\infty \right)=\gamma_{2}\int_{0}^{\infty }I_{2}\text{d}t$ and $\xi \left(\infty \right)=\beta \int_{0}^{\infty }I_{1}\;\text{d}t$. It is straightforward to see that $I_{2}\left(0\right)=0$ and $I_{2}\left(\infty \right)=0$, so integrating the equation for ${\dot{I}}_{2}$ yields $\gamma_{1}\int_{0}^{\infty }I_{1}\;\text{d}t=\gamma_{2}\int_{0}^{\infty }I_{2}\;\text{d}t$. We conclude

$$\xi \left(\infty \right)=\frac{\beta }{\gamma_{1}}R\left(\infty \right)=\frac{\beta }{\gamma_{1}}\left[1-S\left(\infty \right)\right]=\frac{\beta }{\gamma_{1}}\left[1-\left(1-\rho \right)e^{-\xi \left(\infty \right)}\psi \left(\theta \left(\infty \right)\right)\right]\;.$$

We similarly find θ(∞). We have $\theta \left(\infty \right)=\phi_{S}\left(\infty \right)+\phi_{R}\left(\infty \right)$, and $\phi_{S}\left(\infty \right)$ is given above. Thus $\phi_{R}\left(\infty \right)=\theta \left(\infty \right)-\left(1-\rho \right)e^{-\xi \left(\infty \right)}\frac{\psi^{\prime }\left(\theta \left(\infty \right)\right)}{\langle K\rangle }$. Integrating the equation for $\dot{\theta }$ yields

$$\theta \left(\infty \right)=1-\tau_{1}\int_{0}^{\infty }\phi_{I,1}\text{d}t-\tau_{2}\int_{0}^{\infty }\phi_{I,2}\text{d}t\;.$$

We attack the integrals sequentially. We have ${\dot{\phi }}_{R}+{\dot{\phi }}_{I,2}=\gamma_{1}\phi_{I,1}-\tau_{2}\phi_{I,2}$. Integrating from 0 to ∞ and noting $\phi_{I,2}\left(0\right)=\phi_{I,2}\left(\infty \right)=0$, we conclude

$$\gamma_{1}\int_{0}^{\infty }\phi_{I,1}\text{d}t=\phi_{R}\left(\infty \right)+\tau_{2}\int_{0}^{\infty }\phi_{I,2}\text{d}t.$$

So we have

$$\theta \left(\infty \right)=1-\frac{\tau_{1}}{\gamma_{1}}\phi_{R}\left(\infty \right)-\left(\frac{\tau_{1}\tau_{2}}{\gamma_{1}}+\tau_{2}\right)\int_{0}^{\infty }\phi_{I,2}\text{d}t\;.$$

Similarly since ${\dot{\phi }}_{R}=\gamma_{2}\phi_{I,2}$ and $\phi_{R}\left(0\right)=0$, we have $\gamma_{2}\int_{0}^{\infty }\phi_{I,2}\text{d}t=\phi_{R}\left(\infty \right)$. Thus

$$\begin{array}{c} \theta \left(\infty \right)=1-\left[\frac{\tau_{1}}{\gamma_{1}}+\frac{\tau_{1}\tau_{2}}{\gamma_{1}\gamma_{2}}+\frac{\tau_{2}}{\gamma_{2}}\right]\phi_{R}\left(\infty \right)\\ =1-\frac{T_{\text{se}}}{1-T_{\text{se}}}\phi_{R}\left(\infty \right)\\ =1-\frac{T_{\text{se}}}{1-T_{\text{se}}}\left[\theta \left(\infty \right)-\frac{\left(1-\rho \right)e^{-\xi \left(\infty \right)}\psi^{’}\left(\theta \left(\infty \right)\right)}{\langle K\rangle }\right]\;. \end{array}$$

Rearranging this final expression, we conclude

$$\begin{array}{l} \xi \left(\infty \right)=\frac{\beta }{\gamma_{1}}\left[1-\left(1-\rho \right)e^{-\xi \left(\infty \right)}\psi \left(\theta \left(\infty \right)\right)\right]\\ \theta \left(\infty \right)=1-T_{\text{se}}+T_{\text{se}}\frac{\left(1-\rho \right)e^{-\xi \left(\infty \right)}\psi^{’}\left(\theta \left(\infty \right)\right)}{\langle K\rangle }\;. \end{array}$$

We can solve this system iteratively and substitute the result into our expression for S(∞) to find the final size of the epidemic.

##### 2.2.2.2 Direct derivation

It turns out we can derive this directly: We interpret θ(∞) as the probability that a sexual partner either would never transmit even if infected ($1-T_{\text{se}}$), or would transmit, but is never infected ($T_{\text{se}}\frac{\left(1-\rho \right)e^{-\xi \left(\infty \right)}\psi^{\prime }\left(\theta \left(\infty \right)\right)}{\langle K\rangle }$). Adding these yields the expression above. We can similarly derive ξ(∞). This is interpreted as the expected number of mass action transmissions an individual receives. The expected number caused by each infected individual is $\beta /\gamma_{1}$. So $\xi \left(\infty \right)=\beta \left(1-S\left(\infty \right)\right)/\gamma_{1}$. Substituting for *S*, we have

$$\xi \left(\infty \right)=\frac{\beta }{\gamma_{1}}\left[1-\left(1-\rho \right)e^{-\xi \left(\infty \right)}\psi \left(\theta \left(\infty \right)\right)\right]$$

and thus we have a direct derivation of the consistency relation for the final size.

### 2.3 Vector-borne transmission

We now consider transmission with a vector component, which we will refer to as a “mosquito”. We assume there is no latent phase: when an individual or a mosquito is infected it is immediately infectious. We again assume two infectious phases in humans, with human to mosquito transmission possible only in the first phase. We make the same assumptions as before about sexual transmission.

We assume mosquito lifetimes are shorter than epidemic duration. We assume the mosquito population is always close to equilibrium, but more realistic dynamics could be added. Once infected, a mosquito remains infectious until death. The average lifespan of a mosquito is taken to be 1/δ, corresponding to a death-rate of *δ*. The influx of new mosquitos is at a constant rate *B*, measured in mosquitos per unit time per person. The human to mosquito transmission rate is $\beta_{1}$, and the mosquito to human transmission rate is $\beta_{2}$. We use $V_{S}$ and $V_{I}$ to represent the number of susceptible and infected mosquitos per person. We have

$$\begin{array}{l} {\dot{V}}_{S}=B-\beta_{1}I_{1}V_{S}-\delta V_{S}\\ {\dot{V}}_{I}=\beta_{1}I_{1}V_{S}-\delta V_{I}\;. \end{array}$$

**Fig. 5:**
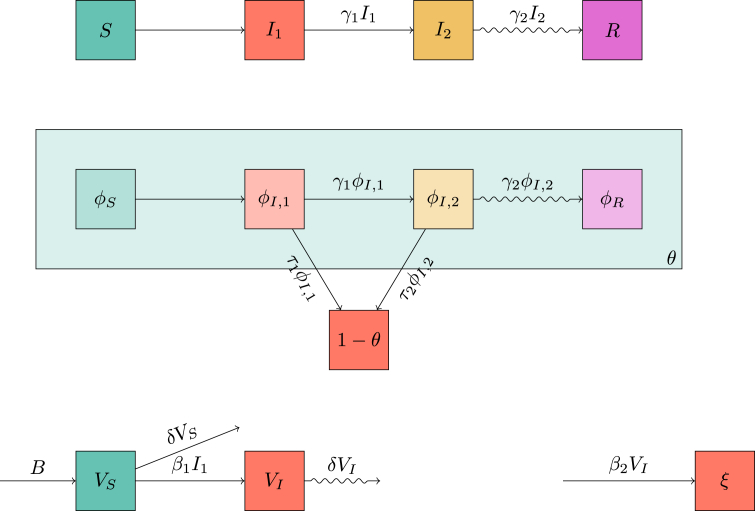
Flow diagrams leading to system (6) for the combined sexual network and vector transmission routes. These are very similar to Fig. 3, except for the introduction of the vector component at the bottom.

Guided by the mixed network-mass-action model, we set *ξ* to be the expected number of transmissions received by a human from mosquitos. Taking other variables as before, and following the flow diagram in Fig. 5, we have

(6a) $$S=\left(1-\rho \right)e^{-\xi }\psi \left(\theta \right)$$

(6b) $$I_{1}=1-S-I_{2}-R$$

(6c) $${\dot{I}}_{2}=\gamma_{1}I_{1}-\gamma_{2}I_{2}$$

(6d) $$\dot{R}=\gamma_{2}I_{2}$$

(6e) $${\dot{V}}_{S}=B-\beta_{1}I_{1}V_{S}-\delta V_{S}$$

(6f) $${\dot{V}}_{I}=\beta_{1}I_{1}V_{S}-\delta V_{I}$$

(6g) $$\dot{\xi }=\beta_{2}V_{I}$$

(6h) $$\dot{\theta }=-\tau_{1}\phi_{I,1}-\tau_{2}\phi_{I,2}$$

(6i) $$\phi_{S}=\left(1-\rho \right)e^{-\xi }\frac{\psi^{\prime }\left(\theta \right)}{\langle K\rangle }$$

(6j) $$\phi_{I,1}=\theta -\phi_{S}-\phi_{I,2}-\phi_{R}$$

(6k) $${\dot{\phi }}_{I,2}=\gamma_{1}\phi_{I,1}-\left(\gamma_{2}+\tau_{2}\right)\phi_{I,2}$$

(6l) $${\dot{\phi }}_{R}=\gamma_{2}\phi_{I,2}\;.$$

**Fig. 6:**
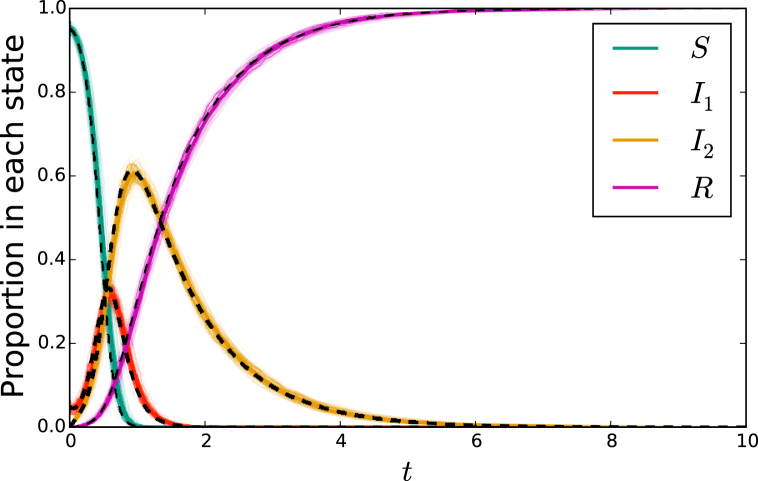
A comparison of simulation with solutions to system (6). Because resolving individual mosquitos significantly increases calculation time, we run fewer calculations with smaller populations. The cloud of simulations consist of 100 simulations in a population of $10^{3}$ individuals with typically 20 mosquitos per individual. Again 3 are highlighted. An additional solid colored curve is given for a population of $10^{4}$ individuals. The dashed black curve represents the solution to the ODEs of system (6). The parameters are δ=1, B=20, $\beta_{1}=\beta_{2}=2$, $\tau_{1}=1$, $\gamma_{1}=5$, $\tau_{2}=0.3$, and $\gamma_{2}=1$, again with $\psi \left(x\right)=\left(x^{2}+x^{4}\right)/2$.

Fig. 6 compares simulated epidemics in large populations with solutions to the equations. We again see excellent fit.

#### 2.3.1 ${ℛ}_{0}$

Because of the similarity to the mass action model we see that $R_{\text{se}|\text{se}}$, the expected number of sexual transmissions from an individual infected through sexual transmission early in the epidemic, is as before. We use $R_{\text{se}|\text{mo}}$ to be the expected number of sexual transmissions caused by an individual infected by mosquitos early in the epidemic. We define $R_{\text{mo}}$ to be the number of human infections resulting from a single infected human through mosquito-based transmission. We assume that the mosquito population is at equilibrium, so $R_{\text{mo}}=\left(B/\delta \right)\left(\beta_{1}/\gamma_{1}\right)\left(\beta_{2}/\delta \right)$. Then ${ℛ}_{0}$ is the leading eigenvalue of

$$\left(\begin{array}{cc} R_{\text{mo}} & R_{\text{mo}}\\ R_{\text{se}|\text{mo}} & R_{\text{se}|\text{se}} \end{array}\right)$$

and so it takes the same form as before. Note that in this definition, we have considered human to mosquito to human to constitute a single generation. An alternate formulation might consider human to mosquito and mosquito to human each as a separate generation. This will lead to a different expression for ${ℛ}_{0}$. See, for example, (Gao et al., 2016). However, the resulting threshold ${ℛ}_{0}=1$ would still be the same.

The fact that ${ℛ}_{0}$ is not simply the sum $R_{\text{mo}}+R_{\text{se}|\text{se}}$ has important implications. Consider a population in which $R_{\text{se}|\text{se}}>1$, but $R_{\text{se}|\text{mo}}<1$, and assume that the vast majority of transmissions are through mosquitos. If we measure the expected number of sexual transmissions an infected individual causes, this will be close to $R_{\text{se}|\text{mo}}$. If we conflate this measured value with $R_{\text{se}|\text{se}}$, then we will underestimate the possibility of a purely sexual epidemic in a population without mosquitos (Allard, Althouse, Hébert-Dufresne, & Scarpino, 2016).

#### 2.3.2 Final size relation

We are not able to find a simple recurrence leading to a finite size relation, and do not believe such a recurrence exists. Generally final size relations can be derived by calculating the number of infections given the number of transmissions occurring, and then relating the number of transmissions occurring given the number of infections (Miller, 2012). In this model such a relation is not possible because the timing of infections matter.

If we assume that all infections happen across a brief window in which there is negligible mosquito turnover, the number of infected mosquitos is bounded by the equilibrium mosquito population size: many mosquitos might bite multiple infected individuals, and many bites have no effect. However, if the epidemic is much slower, then this blocking mechanism is reduced, and so the total number of mosquitos infected may be larger, thus causing a different number of total human infections. It would be possible to determine the number of humans infected if the total number of infected mosquitos is known.

By appropriately changing the parameters of the mosquitos, we could keep $R_{\text{mo}}$ unchanged, but still alter the total number of infected mosquitos, thus altering the total number of infected humans. The usual techniques to derive a final size relation will fail.

### 2.4 Multi-layer networks

We now consider a population in which the potentially transmitting contacts occur within one of two networks. The individuals in the networks are the same, but the partnerships have different interpretations. The first network is the network of sexual partnerships, while the second is a network of social interactions. As before we assume two infectious stages. The first infectious stage transmits with rate $\tau_{1}$ to sexual partners and *β* to social partners. The second stage transmits with rate $\tau_{2}$ to sexual partners and not at all to social partners.

We assume a joint degree distribution $P\left(k_{\text{se}},k_{\text{so}}\right)$, allowing the number of sexual partners to be correlated with the number of social partners, and we define $\psi \left(x,y\right)=\sum_{k_{\text{se}},k_{\text{so}}}P\left(k_{\text{se}},k_{\text{so}}\right)x^{k_{\text{se}}}y^{k_{\text{so}}}$. We define $\theta_{\text{se}}$ to be the probability a sexual partner has not transmitted to an individual *u* and $\theta_{\text{so}}$ to be the probability a social partner has not transmitted to *u* (ignoring the possibility of *u* transmitting to its partners). As before we use $\phi_{S}^{\text{se}}$, $\phi_{I,1}^{\text{se}}$, $\phi_{I,2}^{\text{se}}$, $\phi_{R}^{\text{se}}$ and $\phi_{S}^{\text{so}}$, $\phi_{I,1}^{\text{so}}$, and $\phi_{R}^{\text{so}}$ with the superscript denoting the type of partnership and the subscript denoting the status of the partner. As the second infected state does not cause social infections, we lump it in with *R* for social infections, and write $\phi_{I,1}^{\text{so}}$ as simply $\phi_{I}^{\text{so}}$.

**Fig. 7:**
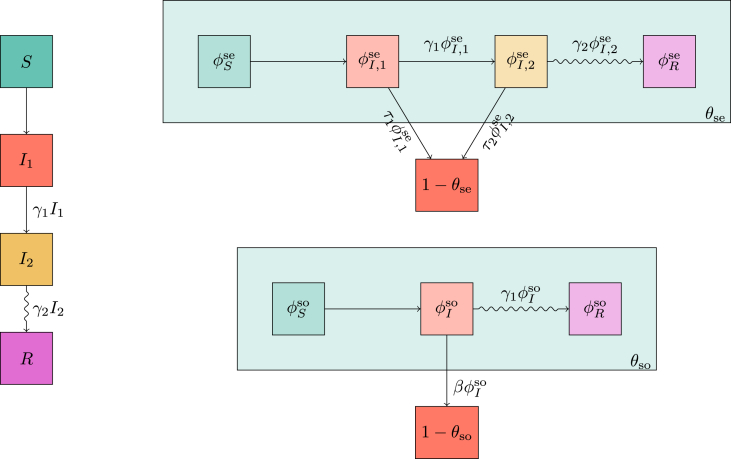
Flow diagrams leading to system (7) for a population in which interactions all lie within a sexual or a social network. We assume that the infectious stage can be subdivided into two stages, the first of which transmits through either contact, and the second transmits only through sexual contact.

The diagrams in Fig. 7 give

(7a) $$S=\left(1-\rho \right)\psi \left(\theta_{\text{se}},\theta_{\text{so}}\right)$$

(7b) $$I_{1}=1-S-I_{2}-R$$

(7c) $${\dot{I}}_{2}=\gamma_{1}I_{1}-\gamma_{2}I_{2}$$

(7d) $$\dot{R}=\gamma_{2}I_{2}$$

(7e) $${\dot{\theta }}_{\text{se}}=-\tau_{1}\phi_{I,1}^{\text{se}}-\tau_{2}\phi_{I,2}^{\text{se}}$$

(7f) $$\phi_{I,1}^{\text{se}}=\theta -\left(1-\rho \right)\frac{\psi_{x}\left(\theta_{\text{se}},\theta_{\text{so}}\right)}{\langle K_{\text{se}}\rangle }-\phi_{I,2}^{\text{se}}-\phi_{R}^{\text{se}}$$

(7g) $${\dot{\phi }}_{I,2}^{\text{se}}=\gamma_{1}\phi_{I,1}^{\text{se}}-\left(\gamma_{2}+\tau_{2}\right)\phi_{I,2}^{\text{se}}$$

(7h) $${\dot{\phi }}_{R}^{\text{se}}=\gamma_{2}\phi_{I,2}^{\text{se}}$$

(7i) $${\dot{\theta }}_{\text{so}}=-\beta \phi_{I,1}^{\text{so}}$$

(7j) $$\phi_{I,1}^{\text{so}}=\theta -\left(1-\rho \right)\frac{\psi_{y}\left(\theta_{\text{se}},\theta_{\text{so}}\right)}{\langle K_{\text{so}}\rangle }-\phi_{R}^{\text{so}}$$

(7k) $${\dot{\phi }}_{R}^{\text{so}}=\gamma_{1}\phi_{I,1}^{\text{so}}\;.$$

**Fig. 8:**
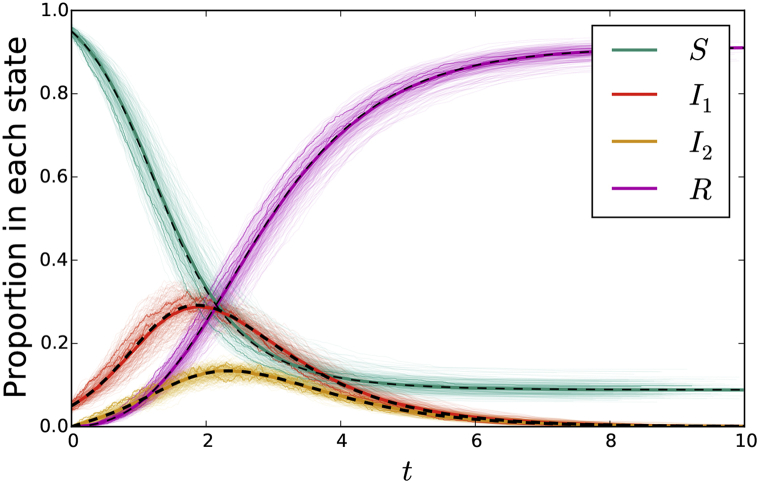
A comparison of simulation with solutions to system (7) for transmission through two overlapping static networks. The cloud consists of simulations in a population of $10^{3}$ individuals, with three simulations highlighted. The other solid colored curves represent a single simulation in a population of $10^{5}$ individuals. The black dashed curve represents the solution to the ODE of system (7). In the sexual network, a quarter of the population has 2 partners, half have 3, and a quarter have 4. In the social network, half have 10 partners and half have 20. Their values are correlated so that the least active individuals in both networks are the same. That is, $\psi \left(x,y\right)=\left(x^{2}y^{10}+x^{3}y^{10}+x^{3}y^{20}+x^{4}y^{20}\right)/4$. The parameters are β=0.1, $\tau_{1}=0.5$, $\gamma_{1}=1$, $\tau_{2}=0.2$, and $\gamma_{2}=2$.

Fig. 8 compares simulated epidemics in large populations, with two networks along which disease transmits. Again the fit is excellent.

#### 2.4.1 ${ℛ}_{0}$

To find ${ℛ}_{0}$ we define $R_{\text{so}|\text{so}}$ to be the expected number of social transmissions caused given that an individual is infected through a social transmission, $R_{\text{so}|\text{se}}$ to be the expected number of social transmissions caused given that an individual is infected through a sexual transmission, and similarly $R_{\text{se}|\text{se}}$ and $R_{\text{se}|\text{so}}$. For notational simplicity, we define $T_{\text{so}}=\beta /\left(\beta +\gamma_{1}\right)$ to be the probability a social contact will transmit before the infected individual moves into the second infectious stage. We again define $T_{\text{se}}=1-\frac{\gamma_{1}}{\tau_{1}+\gamma_{1}}\frac{\gamma_{2}}{\tau_{2}+\gamma_{2}}$ to be the probability a sexual contact will transmit in (at least) one of the two stages. We have

$$\begin{array}{l} R_{\text{se}|\text{se}}=T_{\text{se}}\frac{\psi_{xx}\left(1,1\right)}{\langle K_{\text{se}}\rangle }\\ R_{\text{se}|\text{so}}=T_{\text{se}}\frac{\psi_{xy}\left(1,1\right)}{\langle K_{\text{so}}\rangle }\\ R_{\text{so}|\text{se}}=T_{\text{so}}\frac{\psi_{xy}\left(1,1\right)}{\langle K_{\text{se}}\rangle }\\ R_{\text{so}|\text{so}}=T_{\text{so}}\frac{\psi_{yy}\left(1,1\right)}{\langle K_{\text{so}}\rangle }\;. \end{array}$$

Note that $\psi_{xx}\left(1,1\right)=\langle K_{\text{se}}^{2}-K_{\text{se}}\rangle$, $\psi_{xy}\left(1,1\right)=\langle K_{\text{so}}K_{\text{se}}\rangle$, and $\psi_{yy}\left(1,1\right)=\langle K_{\text{so}}^{2}-K_{\text{so}}\rangle$ Taking $N_{\text{se}}\left(g\right)$ and $N_{\text{so}}\left(g\right)$ to be the number infected by each method in generation *g* we have

$$\left(\begin{array}{c} N_{\text{se}}\left(g+1\right)\\ N_{\text{so}}\left(g+1\right) \end{array}\right)=\left(\begin{array}{cc} R_{\text{se}|\text{se}} & R_{\text{se}|\text{so}}\\ R_{\text{so}|\text{se}} & R_{\text{so}|\text{so}} \end{array}\right)\left(\begin{array}{c} N_{\text{se}}\left(g\right)\\ N_{\text{so}}\left(g\right) \end{array}\right)\;.$$

The dominant eigenvalue of this matrix is ${ℛ}_{0}$.

#### 2.4.2 Final size relation

The final size relation of this model is straightforward to derive directly. Given $\theta_{\text{se}}\left(\infty \right)$ and $\theta_{\text{so}}\left(\infty \right)$, the probability a sexual partner of *u* is never infected is $\left(1-\rho \right)\psi_{x}\left(\theta_{\text{se}}\left(\infty \right),\theta_{\text{so}}\left(\infty \right)\right)/\langle K_{\text{se}}\rangle$. Similarly the probability a social partner of *u* is never infected is $\left(1-\rho \right)\psi_{y}\left(\theta_{\text{se}}\left(\infty \right),\theta_{\text{so}}\left(\infty \right)\right)/\langle K_{\text{so}}\rangle$. Translated into equations, these become

$$\begin{array}{l} \theta_{\text{se}}\left(\infty \right)=1-T_{\text{se}}+T_{\text{se}}\left(1-\rho \right)\frac{\psi_{x}\left(\theta_{\text{se}}\left(\infty \right),\theta_{\text{so}}\left(\infty \right)\right)}{\langle K_{\text{se}}\rangle }\\ \theta_{\text{so}}\left(\infty \right)=1-T_{\text{so}}+T_{\text{so}}\left(1-\rho \right)\frac{\psi_{y}\left(\theta_{\text{se}}\left(\infty \right),\theta_{\text{so}}\left(\infty \right)\right)}{\langle K_{\text{so}}\rangle }\;. \end{array}$$

We can solve this iteratively. From the solution, we have $S\left(\infty \right)=\left(1-\rho \right)\psi \left(\theta_{\text{se}}\left(\infty \right),\theta_{\text{so}}\left(\infty \right)\right)$.

## 3 Models with more complex network structure

We have considered disease spread with a fairly simple network structure combined with various other transmission mechanisms. Now we take the other transmission mechanism to be mass action mixing and consider more complex network structure.

### 3.1 Dynamic network

We begin by assuming that partnerships change in time. We assume the partnership duration is exponentially distributed with mean 1/η. When a partnership ends, the two individuals immediately form new partnerships. Conceptually, we can think of an individual with *k* partners as having *k stubs* (binding sites in the language of (Leung, 2016, Leung et al., 2015)) which connect to stubs of other individuals to form temporary partnerships. Partnerships terminate with rate *η*.

We use a refined definition of *θ*: It is the probability that for a given stub of *u* no partner connected to that stub has ever transmitted to *u*. As before the probability *u* is susceptible is $S\left(t\right)=\left(1-\rho \right)e^{-\xi \left(t\right)}\psi \left(\theta \left(t\right)\right)$, but determining $\phi_{S}$, $\phi_{I}$, and $\phi_{R}$ is different. For this we follow (Kiss et al., 2017, chapter 8).

Let *v* be a random partner of *u* at time *t*. We define ζ(t) to be the probability that the stubs currently forming their partnership have not brought infection to either *u* or *v* prior to joining. Then

$$\phi_{S}=\left(1-\rho \right)e^{-\xi }\zeta \sum_{k_{v}}\frac{k_{v}P\left(k_{v}\right)}{\langle K\rangle }\theta^{k_{v}-1}=\left(1-\rho \right)e^{-\xi }\zeta \frac{\psi^{’}\left(\theta \right)}{\langle K\rangle }\;.$$

The derivative of *ζ* is $\eta \theta^{2}-\eta \zeta$: the rate at which such partnerships are created minus the rate at which such partnerships are destroyed by partnership turnover.

We have an option of how to build the model. We could write $\phi_{S}=\left(1-\rho \right)e^{-\xi }\zeta \frac{\psi^{\prime }\left(\theta \right)}{\langle K\rangle }$ and use a differential equation for *ζ*. However, we choose to use a differential equation for $\phi_{S}$ and eliminate *ζ*. We write

$$\begin{array}{l} {\dot{\phi }}_{S}=-\dot{\xi }\phi_{S}+\dot{\zeta }\left(1-\rho \right)e^{-\xi }\frac{\psi^{’}\left(\theta \right)}{\langle K\rangle }+\dot{\theta }\left(1-\rho \right)e^{-\xi }\zeta \frac{\psi^{’}’\left(\theta \right)}{\langle K\rangle }\\ =-\dot{\xi }\phi_{S}+\eta \theta \pi_{S}-\eta \phi_{S}-\left(\tau_{1}\phi_{I,1}+\tau_{2}\phi_{I,2}\right)\phi_{S}\frac{\psi^{’}’\left(\theta \right)}{\psi^{’}\left(\theta \right)}\;. \end{array}$$

**Fig. 9:**
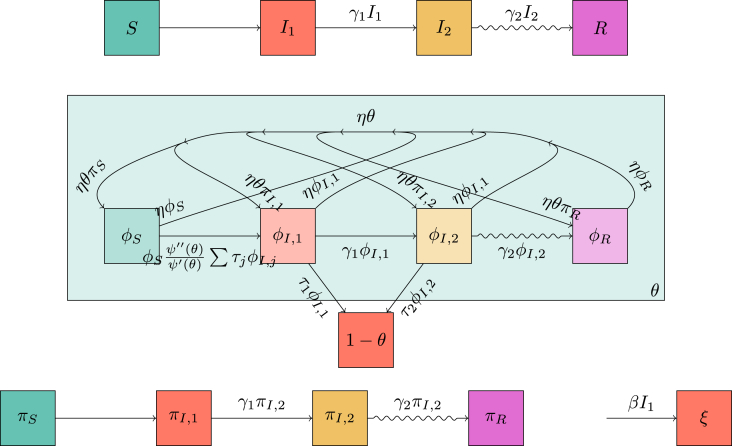
Flow diagrams leading to system (8) for mass action transmission combined with transmission through a dynamic sexual network. The top diagram is as before. The middle diagram is much as before for the network component, but because partnerships can change there are additional paths to take. Because of this we cannot solve for $\phi_{S}$ explicitly and we must calculate all the fluxes in and out of $\phi_{S}$. The fluxes due to finding new partners depend on the probability that the new partner has a given status. These are tracked using the diagram in the bottom left. The bottom right is as before.

We require new variables $\pi_{S}$, $\pi_{I,1}$, $\pi_{I,2}$ and $\pi_{R}$, representing the probability a random stub belongs to an individual of each status. We can directly show that $\pi_{S}=\left(1-\rho \right)\sum_{k}kP\left(k\right)\theta^{k}=\left(1-\rho \right)\theta \psi^{\prime }\left(\theta \right)$. Fig. 9 leads to a new system of equations

(8a) $$S=\left(1-\rho \right)e^{-\xi }\psi \left(\theta \right)$$

(8b) $$I_{1}=1-S-I_{2}-R$$

(8c) $${\dot{I}}_{2}=\gamma_{1}I_{1}-\gamma_{2}I_{2}$$

(8d) $$\dot{R}=\gamma_{2}I_{2}$$

(8e) $$\dot{\xi }=\beta I_{1}$$

(8f) $$\dot{\theta }=-\tau_{1}\phi_{I,1}-\tau_{2}\phi_{I,2}$$

(8g) $${\dot{\phi }}_{S}=-\beta I_{1}\phi_{S}+\eta \theta \pi_{S}-\eta \phi_{S}-\left(\tau_{1}\phi_{I,1}+\tau_{2}\phi_{I,2}\right)\phi_{S}\frac{\psi^{\prime \prime }\left(\theta \right)}{\psi^{\prime }\left(\theta \right)}$$

(8h) $$\phi_{I,1}=\theta -\phi_{S}-\phi_{I,2}-\phi_{R}$$

(8i) $${\dot{\phi }}_{I,2}=\gamma_{1}\phi_{I,1}+\eta \theta \pi_{I,2}-\left(\eta +\gamma_{2}+\tau_{2}\right)\phi_{I,2}$$

(8j) $${\dot{\phi }}_{R}=\eta \theta \pi_{R}+\gamma_{2}\phi_{I,2}-\eta \phi_{R}$$

(8k) $$\pi_{S}=\left(1-\rho \right)\frac{\theta e^{-\xi }\psi^{\prime }\left(\theta \right)}{\langle K\rangle }$$

(8l) $$\pi_{I,1}=1-\pi_{S}-\pi_{I,2}-\pi_{R}$$

(8m) $${\dot{\pi }}_{I,2}=\gamma_{1}\pi_{I,1}-\gamma_{2}\pi_{I,2}$$

(8n) $${\dot{\pi }}_{R}=\gamma_{2}\pi_{I,2}\;.$$

Our initial conditions are

$$\begin{array}{l} I_{2}\left(0\right)=R\left(0\right)=0\\ \theta \left(0\right)=1\\ \phi_{S}\left(0\right)=\left(1-\rho \right)\\ \phi_{I,2}\left(0\right)=\phi_{R}\left(0\right)=0\\ \pi_{I,2}=\pi_{R}=0\\ \xi \left(0\right)=0\;. \end{array}$$

**Fig. 10:**
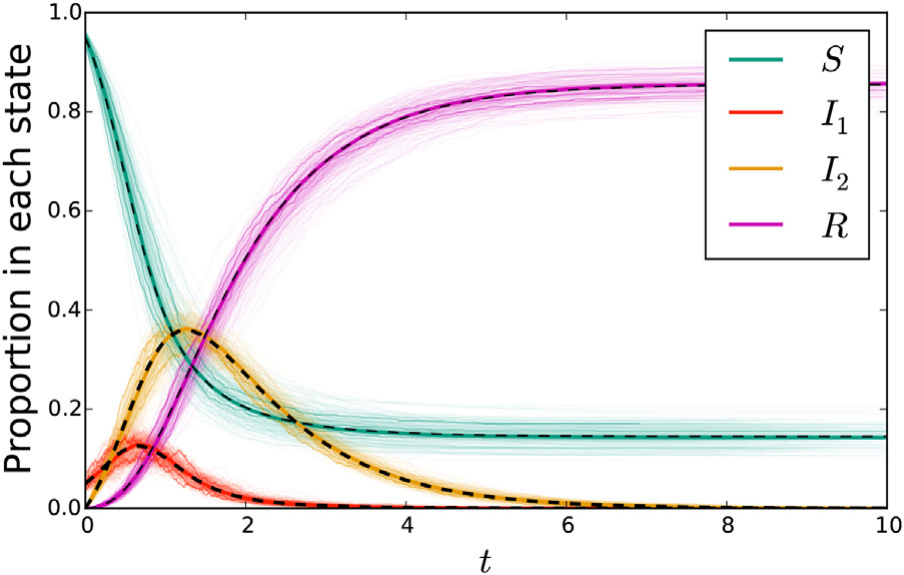
A comparison of simulation with solutions to system (8) for mass action transmission combined with sexual transmission through a dynamic network. The cloud consists of simulations in a population of $10^{3}$ individuals, with three simulations highlighted. The additional solid colored curve is a single simulation in a population of $10^{5}$ individuals. The dashed black curve is the solution to system (8). The parameters are β=5, $\tau_{1}=1$, $\gamma_{1}=5$, $\tau_{2}=0.3$, and $\gamma_{2}=1$. We take $\psi \left(x\right)=\left(x^{2}+2x^{3}+x^{4}\right)/4$.

Fig. 10 compares simulated epidemics in a large population. Transmissions occur through a mass action mechanism and through transmission in a dynamic sexual network.

#### 3.1.1 ${ℛ}_{0}$

Although ${ℛ}_{0}$ can be calculated for this model, the calculation is quite tedious. We must account for the fact that when an SI partnership forms, it can transmit, the partnership can dissolve and be replaced, and retransmit again. It become particularly tedious as we must consider the possible state of the partnership when the first transition happens and calculate different outcomes for whether it is II or SI, and take an appropriate weighted average.

For now, we note that the ${ℛ}_{0}$ calculated for the static network case is a lower bound. We can find an upper bound by assuming the η→∞ limit such the partnerships always change prior to the next transmission. Then when an individual becomes infected along a sexual contact we expect $\langle K^{2}\rangle /\langle K\rangle$ partnerships available to transmit at all times. However when an individual becomes infected through a mass action transmission we expect 〈K〉 partnerships to be available. The number of transmissions occurring for each is $\frac{\tau_{1}}{\gamma_{1}}+\frac{\tau_{2}}{\gamma_{2}}$.

Following the second approach of section 2.2.1 we set N(g) to be the number of individuals infected in generation *g* and y(g) to be the number of partnerships those infected individuals are in. We find

$$N\left(g+1\right)=\frac{\beta }{\gamma_{1}}N\left(g\right)+\left(\frac{\tau_{1}}{\gamma_{1}}+\frac{\tau_{2}}{\gamma_{2}}\right)y\left(g\right)$$

and the number of partnerships they are in is

$$y\left(g+1\right)=\frac{\beta }{\gamma_{1}}\langle K\rangle N\left(g\right)+\left(\frac{\tau_{1}}{\gamma_{1}}+\frac{\tau_{2}}{\gamma_{2}}\right)\frac{\langle K^{2}\rangle }{\langle K\rangle }y\left(g\right)\;.$$

Thus we have defined a matrix problem, and the dominant eigenvalue of

$$\left(\begin{array}{cc} \frac{\beta }{\gamma_{1}} & \frac{\tau_{1}}{\gamma_{1}}+\frac{\tau_{2}}{\gamma_{2}}\\ \frac{\beta }{\gamma_{1}}\langle K\rangle & \left(\frac{\tau_{1}}{\gamma_{1}}+\frac{\tau_{2}}{\gamma_{2}}\right)\frac{\langle K^{2}\rangle }{\langle K\rangle } \end{array}\right)$$

is an upper bound for ${ℛ}_{0}$.

#### 3.1.2 Final size relation

Again we are unable to find a final size relation for much the same reason as in the vector-borne case. The relative timing of the epidemic and the partnership turnover mean that the dynamics of the process play an important role. We cannot express how many transmissions some number of infected individuals will cause because.

### 3.2 Sexual network with preferential mixing

Finally we consider a model in which individuals select their partners preferentially according to degree. This has been studied using pair-approximations (Moreno, Gómez, & Pacheco, 2003), but to our knowledge, the corresponding EBCM model has not been explicitly considered. It can be inferred from model 2.2.3 of (Miller & Volz, 2013), and appears as an exercise in (Kiss et al., 2017, chapter 6). We will study the slightly more general case in which there are two infectious stages as before, and allow for the first infectious stage to also exhibit mass action transmission.

**Fig. 11:**
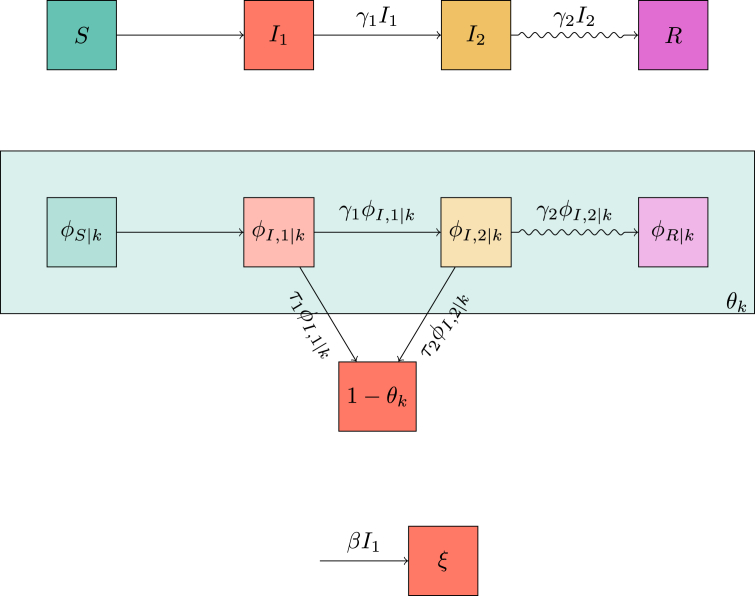
Flow diagrams leading to system (9) for mass action transmission combined with sexual transmission in a preferentially mixing sexual network. The top diagram is as before. The middle diagram tracks $\theta_{k}$ so there is a corresponding equation for each degree *k*. The bottom diagram is as before.

The flow diagrams of Fig. 11 lead to

(9a) $$S=\left(1-\rho \right)e^{-\xi }\sum_{k}P\left(k\right)\theta_{k}^{k}$$

(9b) $$I_{1}=1-S-I_{2}-R$$

(9c) $${\dot{I}}_{2}=\gamma_{1}I_{1}-\gamma_{2}I_{2}$$

(9d) $$\dot{R}=\gamma_{2}I_{2}$$

(9e) $${\dot{\theta }}_{k}=-\tau_{1}\phi_{I,1|k}-\tau_{2}\phi_{I,2|k}$$

(9f) $$\phi_{S|k}=\left(1-\rho \right)e^{-\xi }\sum_{\hat{k}}P_{n}\left(\hat{k}|k\right)\theta_{\hat{k}}^{\hat{k}-1}$$

(9g) $$\phi_{I,1|k}=\theta_{k}-\phi_{S|k}-\phi_{I,2|k}-\phi_{R|k}$$

(9h) $${\dot{\phi }}_{I,2|k}=\gamma_{1}\phi_{I,1|k}-\left(\tau_{2}+\gamma_{2}\right)\phi_{I,2|k}$$

(9i) $${\dot{\phi }}_{R|k}=\gamma_{2}\phi_{I,2|k}$$

(9j) $$\dot{\xi }=\beta I_{1}\;.$$

The initial conditions are

$$\begin{array}{l} I_{2}\left(0\right)=R\left(0\right)=0\\ \theta_{k}\left(0\right)=1\\ \phi_{I,2|k}=\phi_{R|k}=0\\ \xi \left(0\right)=0\;. \end{array}$$

**Fig. 12:**
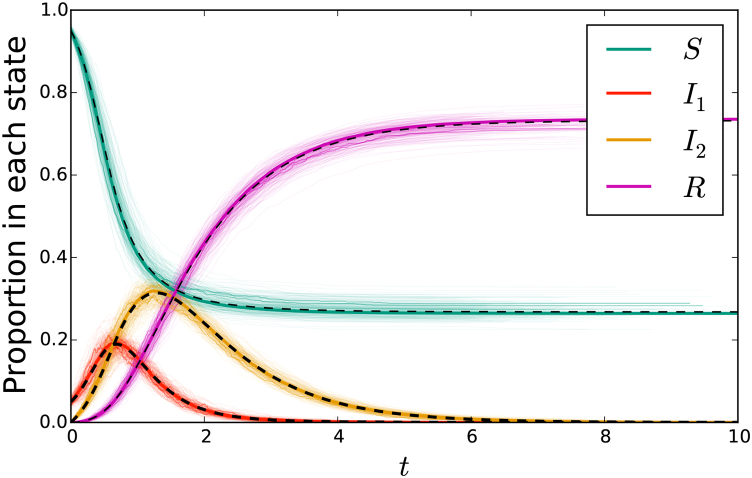
A comparison of simulation with solutions to system (9) for mass action transmission combined with a sexual network exhibiting preferential partnership formation. The cloud represents 200 simulations in a population of $10^{3}$ individuals, with three simulations highlighted. The additional solid colored curve comes from a simulation of $10^{5}$ individuals. The dashed black curve is the solution to system (9). The parameters are given in the text.

Fig. 12 compares simulated epidemics in a large population, with a mass action transmission mechanism and a static sexual network exhibiting preferential mixing. The sexual network has P(1)=P(5)=P(10)=1/3, with the partnerships distributed so that

$$\begin{array}{ccc} P_{n}\left(1|1\right)=1/2 & P_{n}\left(1|5\right)=3/40 & P_{n}\left(1|10\right)=1/80\\ P_{n}\left(5|1\right)=3/8 & P_{n}\left(5|5\right)=1/2 & P_{n}\left(5|10\right)=17/80\\ P_{n}\left(10|1\right)=1/8 & P_{n}\left(10|5\right)=17/40 & P_{n}\left(10|10\right)=62/80 \end{array}$$

As before, the infectious period has two stages. The first infectious stage $I_{1}$ transmits through both mechanisms with $\tau_{1}=1$, β=1, and $\gamma_{1}=3$. The second stage has $\tau_{2}=0.1$ and $\gamma_{2}=1$.

#### 3.2.1 ${ℛ}_{0}$

To find ${ℛ}_{0}$ for mass action transmission combined with sexual transmission on a static preferentially mixing network, we define $N_{k}\left(g\right)$ to be the number of individuals having *k* partners that are infected in generation *g*. We again use $T_{\text{se}}=1-\frac{\gamma_{1}\gamma_{2}}{\left(\tau_{1}+\gamma_{1}\right)\left(\tau_{2}+\gamma_{2}\right)}$ to be the probability that an infected individual transmits to a susceptible partner before recovering. The expected number of infections of individuals with degree $\hat{k}$ caused through sexual transmission by an infected individual with degree *k* is $T_{\text{se}}kP\left(\hat{k}|k\right)$. The expected number of infections of individuals with degree $\hat{k}$ caused through mass action transmission by any infected individual is $P\left(\hat{k}\right)\beta /\gamma_{1}$. Thus we conclude that

$$N_{\hat{k}}\left(g+1\right)=\sum_{k}\left(T_{\text{sekP}}\left(\hat{k}|k\right)+P\left(\hat{k}\right)\frac{\beta }{\gamma_{1}}\right)N_{k}\left(g\right)\;.$$

Writing this as a matrix equation, we have

$$\left(\begin{array}{c} N_{0}\left(g+1\right)\\ N_{1}\left(g+1\right)\\ N_{2}\left(g+1\right)\\ N_{3}\left(g+1\right)\\ \vdots \end{array}\right)=\left[T_{\text{se}}\left(\begin{array}{ccccc} 0 & 0 & 0 & 0 & \cdots \\ 0 & P\left(1|1\right) & 2P\left(1|2\right) & 3P\left(1|3\right) & \cdots \\ 0 & P\left(2|1\right) & 2P\left(2|2\right) & 3P\left(2|3\right) & \cdots \\ 0 & P\left(3|1\right) & 2P\left(3|2\right) & 3P\left(3|3\right) & \cdots \\ \vdots & \vdots & \vdots & \vdots & \ddots \end{array}\right)+\frac{\beta }{\gamma_{1}}\left(\begin{array}{ccccc} P\left(0\right) & P\left(0\right) & P\left(0\right) & P\left(0\right) & \cdots \\ P\left(1\right) & P\left(1\right) & P\left(1\right) & P\left(1\right) & \cdots \\ P\left(2\right) & P\left(2\right) & P\left(2\right) & P\left(2\right) & \cdots \\ P\left(3\right) & P\left(3\right) & P\left(3\right) & P\left(3\right) & \cdots \\ \vdots & \vdots & \vdots & \vdots & \ddots \end{array}\right)\right]\left(\begin{array}{c} N_{0}\left(g\right)\\ N_{1}\left(g\right)\\ N_{2}\left(g\right)\\ N_{3}\left(g\right)\\ \vdots \end{array}\right)$$

Then ${ℛ}_{0}$ is the dominant eigenvalue of the matrix given by the sum within the square brackets.

#### 3.2.2 Final size

Unlike the dynamic case, we can derive a final size relation for the preferential mixing model. This is very similar to our basic combined mass action and network model.

We note that

$$\theta_{k}\left(\infty \right)=\left(1-T_{\text{se}}\right)+T_{\text{se}}\phi_{S|k}\left(\infty \right)=\left(1-T_{\text{se}}\right)+T_{\text{se}}\left(1-\rho \right)e^{-\xi \left(\infty \right)}\sum_{\hat{k}}P_{n}\left(\hat{k}|k\right)\theta_{\hat{k}}^{\hat{k}-1}$$

and

$$\xi \left(\infty \right)=\frac{\beta }{\gamma_{1}}\left[1-S\left(\infty \right)\right]=\frac{\beta }{\gamma_{1}}\left[1-\left(1-\rho \right)e^{-\xi \left(\infty \right)}\sum_{k}P\left(k\right)\theta_{k}^{k}\left(\infty \right)\right]\;.$$

This provides the relation to solve. Once we have this, then

$$R\left(\infty \right)=1-\left(1-\rho \right)e^{-\xi \left(\infty \right)}\sum_{k}P\left(k\right)\theta_{k}^{k}\left(\infty \right).$$

## Footnotes

1 For example, with (4), if we take $\theta_{0}=1$ and $\phi_{S}\left(0\right)=1-\rho$, then iteration directly gives us $\theta_{1}$, the value after one generation from which we can infer the number infected in the first generation. Repeating, we can capture the entire dynamics.
